# Altered Ca^2+^ Handling and Oxidative Stress Underlie Mitochondrial Damage and Skeletal Muscle Dysfunction in Aging and Disease

**DOI:** 10.3390/metabo11070424

**Published:** 2021-06-28

**Authors:** Antonio Michelucci, Chen Liang, Feliciano Protasi, Robert T. Dirksen

**Affiliations:** 1 DNICS, Department of Neuroscience, Imaging, and Clinical Sciences, University G. d’Annunzio of Chieti-Pescara, I-66100 Chieti, Italy; 2 Department of Pharmacology and Physiology, School of Medicine and Dentistry, University of Rochester Medical Center, Rochester, NY 14642, USA; Chen_Liang@URMC.Rochester.edu (C.L.); Robert_Dirksen@URMC.Rochester.edu (R.T.D.); 3 CAST, Center for Advanced Studies and Technology, DMSI, Department of Medicine and Aging Sciences, University G. d’Annunzio of Chieti-Pescara, I-66100 Chieti, Italy; feliciano.protasi@unich.it

**Keywords:** Ca^2+^ signaling, mitochondria, oxidative stress, skeletal muscle disease, atrophy, sarcopenia

## Abstract

Skeletal muscle contraction relies on both high-fidelity calcium (Ca^2+^) signals and robust capacity for adenosine triphosphate (ATP) generation. Ca^2+^ release units (CRUs) are highly organized junctions between the terminal cisternae of the sarcoplasmic reticulum (SR) and the transverse tubule (T-tubule). CRUs provide the structural framework for rapid elevations in myoplasmic Ca^2+^ during excitation–contraction (EC) coupling, the process whereby depolarization of the T-tubule membrane triggers SR Ca^2+^ release through ryanodine receptor-1 (RyR1) channels. Under conditions of local or global depletion of SR Ca^2+^ stores, store-operated Ca^2+^ entry (SOCE) provides an additional source of Ca^2+^ that originates from the extracellular space. In addition to Ca^2+^, skeletal muscle also requires ATP to both produce force and to replenish SR Ca^2+^ stores. Mitochondria are the principal intracellular organelles responsible for ATP production via aerobic respiration. This review provides a broad overview of the literature supporting a role for impaired Ca^2+^ handling, dysfunctional Ca^2+^-dependent production of reactive oxygen/nitrogen species (ROS/RNS), and structural/functional alterations in CRUs and mitochondria in the loss of muscle mass, reduction in muscle contractility, and increase in muscle damage in sarcopenia and a wide range of muscle disorders including muscular dystrophy, rhabdomyolysis, central core disease, and disuse atrophy. Understanding the impact of these processes on normal muscle function will provide important insights into potential therapeutic targets designed to prevent or reverse muscle dysfunction during aging and disease.

## 1. Ca^2+^ Signaling in Skeletal Muscle

Calcium (Ca^2+^) is a universal second messenger used by virtually all mammalian cells to control a wide range of physiological/biological processes including differentiation, apoptosis, gene transcription, migration, excitability, neurotransmitter secretion, and muscle contraction. Ca^2+^ signaling occurs when the free cytosolic Ca^2+^ concentration, maintained around 100 nM under resting conditions, rises rapidly upon Ca^2+^ release from intracellular stores and/or Ca^2+^ entry from the extracellular space, due to the combination of the opening of Ca^2+^-permeable channels and a steep concentration gradient (~4 orders of magnitude).

### 1.1. Excitation–Contraction (EC) Coupling in Skeletal Muscle

**Figure 1:**
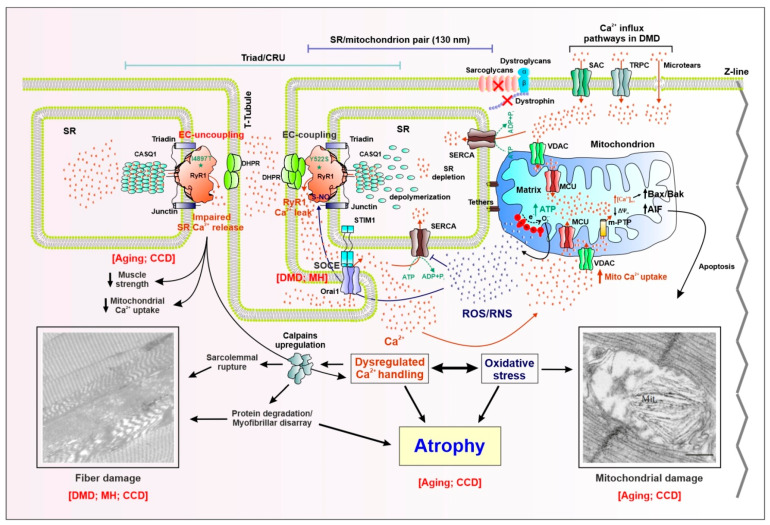
Schematic model showing proposed molecular mechanisms for altered Ca^2+^ signaling, mitochondrial function, and muscle fiber damage/atrophy in skeletal muscle disease and aging. Skeletal muscle contraction relies on a rapid and massive release of Ca^2+^ from the sarcoplasmic reticulum (SR) terminal cisternae upon depolarization of the sarcolemma. Excitation–contraction (EC) coupling, the process whereby an action potential in the surface membrane is converted into Ca^2+^ release from SR, is mediated by a functional coupling between the dihydropyridine receptor (DHPR) voltage sensor in the transverse tubule (T-tubule) membrane and the type-1 ryanodine receptor (RyR1) Ca^2+^ release channel in the SR. The fundamental structure that mediates EC coupling is the Ca^2+^ release unit (CRU), or “triad”, which is composed of a central T-tubule flanked by adjacent junctions with two SR terminal cisternae. Besides DHPR and RyR1, other proteins participate in EC coupling: triadin and junction in the SR membrane, and calsequestrin-1 (CASQ1), the Ca^2+^-binding protein resident in the SR lumen. The ATP needed for muscle contraction is primarily generated within the mitochondria during aerobic respiration. In fast-twitch fibers, most mitochondria are located on the Z-line side of the triad, closely associated with the terminal SR cisternae via small (~8–10 nm) electron dense bridges termed “tethers”. As a result of this structural linkage, the average minimal distance between the RyR1 (site of Ca^2+^ release during EC coupling) and the outer membrane of the adjacent mitochondrion is ~130 nm. Right: Myoplasmic Ca^2+^ overload is the result of: (i) excessive SR Ca^2+^ release, due to gain-of-function point mutations (e.g., Y522S) in RyR1 linked to muscle disorders such as malignant hyperthermia (MH) and central core disease (CCD), that enhance channel opening probability; (ii) enhanced Ca^2+^ influx via STIM1/Orai1-dependent store-operated Ca^2+^ entry (SOCE), as the result of reduced SR Ca^2+^ content (SR depletion), stretch-activated Ca^2+^ channels (SAC), transient receptor potential canonical channels (TRPC), and microtears. These Ca^2+^ influx mechanisms are upregulated in Duchenne muscular dystrophy (DMD) and MH (SOCE). The resulting increase in Ca^2+^ influx into the myoplasm promotes mitochondrial Ca^2+^ uptake through the mitochondrial Ca^2+^ uniporter (MCU), ultimately leading to mitochondrial Ca^2+^ overload that increases electron transport chain activity and excessive production of reactive oxygen and nitrogen species (ROS/RNS), which underlie oxidative stress. In turn, increased ROS/RNS levels oxidize/nytrosylate both RyR1, which further enhances SR Ca^2+^ release channel opening, and the sarco/endoplasmic Ca^2+^ ATPase (SERCA), which reduces SR Ca^2+^ reuptake. The resulting accumulation of Ca^2+^ in the myoplasm, together with increased oxidative stress, triggers a series of intracellular signaling pathways (e.g., calpains activation, reduced protein synthesis, and increased protein degradation) that lead to: (i) myofibrillar disarray, (ii) sarcolemmal rupture, (iii) structural alterations (e.g., contractures, *cores*), and (iv) mitochondrial damage. These alterations, together with increased apoptosis, triggered by mitochondrial Ca^2+^ overload via the activation of the Bax/Bak/AIF pathway, drive loss of muscle mass and atrophy. Left: EC uncoupling, due to the reduction of DHPR expression during aging or as the result of RyR1 loss-of-function point mutations (e.g., I4897T) linked to myopathies such as CCD, reduces electricallyevoked SR Ca^2+^ release that contributes to reduced muscle specific force production, disrupted mitochondrial structure/function, mitochondrial damage, and fiber structural alterations (e.g., formation of *cores* and myofibrillar disarray). The two pictures showing fiber and mitochondrial damage are modified from *Michelucci et al., 2017 Oxid Med Cell Longev. 2017; 2017: 6936897,* and Michelucci et al., *2017 Oxid Med Cell Longev. 2017; 2017: 6792694,* respectively.

Muscle contraction and relaxation are regulated by rapid changes of myoplasmic Ca^2+^. To accomplish this task, skeletal muscle fibers utilize a highly organized sarcotubular membrane system consisting of a dense network of specialized invaginations of the sarcolemma, termed transverse tubules (T-tubules), and a continuous system of internal membranes of sarcoplasmic reticulum (SR). The SR is composed of two distinct functional and morphological compartments in direct continuity with each other: (1) the SR terminal cisternae and (2) longitudinal or free SR [1,2]. The Ca^2+^ release unit (CRU), or “triad”, is composed of a central T-tubule flanked by adjacent junctions with two SR terminal cisternae [3] (Figure 1). The CRU is the fundamental structure that mediates excitation-contraction (EC) coupling, a mechanism whereby an action potential in the T-tubule membrane is converted into a rapid and massive increase of myoplasmic Ca^2+^ concentration. In skeletal muscle, EC coupling involves a unique physical or mechanical interaction between two different types of Ca^2+^ channels: voltage-gated L-type Ca^2+^ channels (CaV1.1) or dihydropiridine receptors (DHPRs), which function as voltage sensors in the T-tubule membrane, and type-1 ryanodine receptor (RyR1) Ca^2+^ release channels in the SR [4,5,6,7] (Figure 1). Following depolarization of the T-tubule membrane, DHPR voltage sensor proteins undergo a conformational change that is mechanically coupled to the opening of nearby RyR1 Ca^2+^ release channels [8,9,10,11]. The subsequent rapid and massive release of Ca^2+^ from the SR into the myoplasm provides the chemical signal used to drive muscle contraction. The high amount of free (~300–500 µM) and bound Ca^2+^ within the SR [12,13,14], coupled with steep gradient across the SR membrane, is achieved by two fundamental proteins that work in concert: calsequestrin-1 (CASQ1) and sarco/endoplasmic Ca^2+^ ATPases (SERCA). CASQ1, a highly acidic protein resident in the SR terminal cisternae, has two major roles during EC coupling: (1) to bind/buffer a large amount of Ca^2+^ needed for release and activation of muscle contraction [15,16,17,18] and (2) to regulate RyR1 activity by reducing channel open probability during SR Ca^2+^ depletion [19,20,21,22]. SERCA is a high-affinity, ATP-dependent Ca^2+^ pump densely packed in the free SR [23,24]. SERCA-mediated SR Ca^2+^ reuptake is the primary mechanism for Ca^2+^ clearance in skeletal muscle following release during EC coupling, allowing muscle relaxation [23,25]. In fact, it is estimated that ~90% of the Ca^2+^ increase during a single twitch contraction (e.g., the SR release induced by a single action potential) is cleared from the myoplasm through SERCA pumps. As a result, SERCA-mediated Ca^2+^ reuptake is responsible for muscle relaxation and recovery of SR Ca^2+^ to levels needed for subsequent cycles of EC coupling.

### 1.2. Store-Operated Ca^2+^ Entry in Skeletal Muscle

DHPR/RyR1-mediated EC coupling and muscle contraction has long been known to persist in the absence of extracellular Ca^2+^ [26] and extracellular Ca^2+^ does not play a critical role in mechanical EC coupling in skeletal muscle [4,27,28,29]. Over the past two decades, however, a growing body of evidence indicates that influx of external Ca^2+^ into muscle fibers plays an important role both in muscle development/growth and in the maintenance of Ca^2+^ release and force generation during repetitive stimulation. Indeed, between the late 1990s and early 2000s, a robust store-operated Ca^2+^ entry (SOCE) pathway was unequivocally identified in both skeletal myotubes [30] and adult muscle fibers [31]. SOCE, a Ca^2+^ influx pathway activated by depletion of intracellular Ca^2+^ stores, is among the most important Ca^2+^ influx pathways in non-excitable cells [32,33,34]. SOCE is coordinated by a functional interaction between stromal-interacting molecule-1 (STIM1), the ER/SR luminal Ca^2+^ sensor [35,36,37], and ORAI1, the Ca^2+^ permeable channel of the plasma membrane [38,39,40]. Coupling of STIM1 and ORAI1 during SR Ca^2+^ depletion also serves as the primary molecular machinery of SOCE in skeletal muscle [41]. Although still not fully understood, intensive research over the past two decades has revealed several important aspects of the molecular mechanism and functional role of SOCE in skeletal muscle. First, SOCE plays a role in refilling SR Ca^2+^ stores, needed to sustain Ca^2+^ release and force generation during repetitive, high-frequency stimulation [42,43,44,45,46]. SOCE also plays a role in fatigue-resistant type I specification during postnatal development [47,48]. Together, these effects of SOCE serve to mitigate muscle susceptibility to fatigue during prolonged activity. Second, acute exercise drives the formation of new junctions within the I band of the sarcomere between SR-stacks and extension of T-tubule. These exercise-induced SR-T-tubule junctions are structurally distinct from triads as they lack RyR1 and DHPR proteins, but contain co-localized STIM1 and ORAI1 proteins that mediate SOCE, and thus, are referred to as “Ca^2+^ entry units” (CEUs) [44,45,46,49]. CEUs are also present, though fewer in number, in resting (non-exercised) muscle. Limited co-localization of STIM1 and ORAI1 at the triad [42,47] together with the presence of some CEUs in close proximity of the triad under resting (non-exercised) conditions [44] provides the machinery needed for the rapid activation of SOCE shown in skeletal muscle [50,51,52,53].

Exercise-induced assembly of additional CEUs, which requires remodeling of SR and of T-tubules at the I band, likely reflects the increased need to replenish intracellular SR Ca^2+^ stores during repetitive stimulation. Interestingly, we found that a key prerequisite for exercise-induced assembly of CEUs and increased SOCE function involves elongation of the T-tubule (containing Orai1) and association with stacks of SR membranes (containing STIM1) within the I band of the sarcomere. Assembly of CEUs following exercise provides a greater surface area for increased STIM1/Orai1 coupling needed to enhance Ca^2+^ influx during sustained muscle activity [44,45,46].

Besides being important in the physiology of adult muscle fibers, SOCE is also critical for the correct development and maturation of skeletal muscle tissue [54,55]. This is demonstrated by the fact that normal muscle development and fiber type specification are altered by early developmental muscle-specific ablation of STIM1 [56], ORAI1 [48], and muscle-specific expression of a dominant-negative ORAI1 [42]. Moreover, a growing body of evidence indicates that loss- and gain-of-function mutations in both STIM1 and ORAI1 are linked to multiple human diseases in which myopathy is a prominent clinical manifestation [57,58]. In addition, dysfunctional or enhanced STIM1/ORAI1-mediated SOCE is also implicated in the pathogenesis of other muscle disorders including muscular dystrophy [59,60,61], malignant hyperthermia [62], and sarcopenia [63,64]. Together, these findings demonstrate that a tight regulation of STIM1/ORAI1-dependent SOCE is critical for optimal muscle performance and that aberrant SOCE function contributes to muscle disease.

## 2. Mitochondria in Skeletal Muscle

### 2.1. Mitochondrial ATP Production and ROS Generation

In addition to Ca^2+^, muscle contraction also requires energy from ATP [65]. ATP hydrolysis is the universal biochemical reaction that provides energy to support a wide range of cellular processes including biosynthesis, active ion transport across membranes, and crossbridge cycling during muscle contraction. ATP is produced from multiple sources in skeletal muscle fibers. Rapid ATP generation occurs in the cytoplasm during hydrolysis of phosphocreatine and glycolysis. However, these anaerobic metabolic pathways provide only a small fraction of total ATP produced in skeletal muscle. The majority of ATP is generated within the mitochondria during a series of reactions that require molecular oxygen (O_2_) in a process referred to as aerobic respiration [66,67,68]. The transfer of electrons from NADH and FADH_2_, through a series to reactions to molecular O_2_ as the final electron acceptor, produces an electrochemical proton gradient across the inner mitochondrial membrane that is used to drive ATP generation by the F_1_/F_0_ ATP synthase. Under normal conditions, only ~1% of electrons that flow through the electron transport chain “slip” to molecular O_2_ to produce superoxide anions (O_2_^•−^). Either spontaneously or in a reaction catalyzed by superoxide dismutases (SODs), O_2_^•−^ is converted to hydrogen peroxide (H_2_O_2_). Together, O_2_^•−^, H_2_O_2_, and hydroxyl radical (HO^•^) are collectively referred to as reactive oxygen species (ROS). Besides mitochondria, other cellular sources of ROS include extra-mitochondrial enzymes such as NADPH oxidases or xanthine oxidase. While for many years ROS produced as a byproduct of the mitochondrial metabolism were only considered pathogenic, recent evidence indicates that low physiological levels of mitochondrial ROS play an important role in cellular redox signaling pathways that regulate multiple cellular functions [69,70]. However, excessive ROS generation, often due to mitochondrial dysfunction, can lead to destructive or pathogenic levels of oxidative stress that contribute to muscle dysfunction in muscle disease (detailed in Section 3, Section 4 and Section 5 below).

### 2.2. Mitochondrial Ca^2+^ Uptake and Ca^2+^ Microdomains in Skeletal Muscle

The hypothesis that mitochondria regulate intracellular Ca^2+^ homeostasiswas proposed in early studies that showed robust Ca^2+^ uptake by purified rat kidney mitochondria [71]. The driving force for accumulation of Ca^2+^ within the mitochondrial matrix is provided by the large negative electrochemical gradient (>−180 mV) across the mitochondrial inner membrane generated by electron transfer chain dependent H^+^ pumping during oxidative phosphorylation. Nonetheless, the affinity of mitochondria for Ca^2+^ uptake is relatively low, with a Ca^2+^ concentration required for half-maximal Ca^2+^ transport of ~30 µM [72]. This concentration is considerably higher than that measured within the muscle fibers not only at rest (~100 nM), but even at the peak of the global myoplasmic Ca^2+^ transient (1–3 µM) during activation of skeletal muscle contraction [73,74]. These observations raised questions regarding the potential physiological relevance of mitochondrial Ca^2+^ uptake in skeletal muscle. However, with the development of mitochondria-targeted Ca^2+^ sensitive probes [75], mitochondrial Ca^2+^ uptake during cytoplasmic Ca^2+^ oscillations was confirmed in multiple cell types (e.g., fibroblasts, neurons, endothelial cells) including skeletal muscle fibers [76,77]. The increase in cytosolic Ca^2+^ promotes the opening of the mitochondrial Ca^2+^ uniporter (MCU), a 40-kDa protein that functions as a highly Ca^2+^-selective channel within the mitochondrial inner membrane, thus enabling Ca^2+^ flux down the high electrochemical gradient from the cytoplasm to the mitochondrial matrix [78,79]. The entry of Ca^2+^ within the mitochondrial matrix stimulates the activity of several enzymes of the tricarboxylic acid (TCA) cycle and electron transport chain including pyruvate dehydrogenase, isocitrate dehydrogenase, α-ketoglutarate dehydrogenase, and the ATP synthetic activity of the F_1_/F_0_ ATPase [80]. As a result, mitochondria both shape the myoplasmic Ca^2+^ transient [81] and are energetically coupled to the metabolic needs of muscle through Ca^2+^-mediated regulation of mitochondrial TCA and electron transport chain activity. The essential role of Ca^2+^ in regulating mitochondrial function in skeletal muscle is further highlighted by the severe clinical muscle phenotypes (e.g., severe muscle weakness, fatigue, lethargy) observed in patients with loss-of-function mutations in MICU1, a modulatory subunit of the MCU complex that acts as a gatekeeper for mitochondrial Ca^2+^ entry [82,83,84].

How mitochondria in skeletal muscle are able to take up Ca^2+^ ions when the peak myoplasmic Ca^2+^ level is an order of magnitude lower than the concentration for half-maximal Ca^2+^ transport by MCU, is still debated. This apparent paradox is explained by a “Ca^2+^ microdomain” that results from the close positioning of mitochondria relative to sites of Ca^2+^ release (i.e., triads or CRUs in skeletal muscle). The concept of a Ca^2+^ microdomain suggests that mitochondrial Ca^2+^ uptake is driven by a high local concentration of Ca^2+^ around mitochondria that is much higher than that observed in the bulk myoplasm [85]. In skeletal fibers, mitochondria can be divided into three main classes: (a) longitudinal mitochondria, which form in rows between myofibrils (found mostly during maturation and in oxidative, slow-twitch fibers); (b) subsarcolemmal mitochondria, located in clusters under the surface membrane in proximity of capillaries (hence, also more frequently observed in more vascularized slow-twitch fibers); and (c) transversal mitochondria, which form in two transverse rows on both sides of Z-lines encircling myofibrils at the I band. The first description of a population of inter-myofibrillar mitochondria positioned within the I band of the sarcomere on both sides of the Z-line was made from elegant electron microscopy (EM) studies conducted by Ogata and Yamasaki [86,87,88,89]. Subsequent quantitative analyses of slow- and fast-twitch muscle fibers showed that mitochondria are preferentially oriented in transverse double rows with a sarcomeric periodicity of ~2 µm, consistent with their close apposition to triads [90]. 

More recently, EM studies in fast-twitch fibers from adult mice showed that:(a) mitochondrial positioning closely follows maturation of the EC coupling system, changing from predominantly longitudinal to transversal positioning during postnatal development; (b) in adult muscle fibers, most mitochondria are located on the Z-line side of the triad, closely associated with the terminal SR cisternae via small (~8–10 nm) electron dense bridges termed “tethers” (Figure 1). The tethers anchor the mitochondrial outer membrane to the SR, thus limiting/preventing mitochondrial movement from sites of Ca^2+^ release. As a result of this structural linkage, the average minimal distance between the RyR1 foot (site of Ca^2+^ release during EC coupling) and the outer membrane of the adjacent mitochondrion is only ~130 nm [91], thus creating a tightly coupled Ca^2+^ signaling SR-mitochondrial “nanodomain” (Figure 1). The CRU–mitochondrial nanodomain could represent the fundamental structural feature required to create the high Ca^2+^ microdomain needed to drive mitochondrial Ca^2+^ uptake via MCU [92] (Figure 1). Consistent with a critical role of this association for Ca^2+^ signaling between the two organelles, the frequency of osmotic shock-induced Ca^2+^ sparks is reduced three-fold during postnatal development, in direct linear correspondence with an increase in mitochondrion–CRU pairing [77]. Moreover, a reduction in CRU/mitochondria tethering was shown to contribute to impaired Ca^2+^ signaling, an increase in mitochondrial-dependent oxidative stress, and reduced muscle performance during aging [93]. These findings are discussed in greater detail in Section 4.

Precise regulation of Ca^2+^ signaling and ROS production is a fundamental pre-requisite for the correct function of skeletal muscle. Consistent with this, multiple pathological conditions in muscle are linked to dysregulation of Ca^2+^ homeostasis and/or Ca^2+^-dependent oxidative stress. In the following three sections, we discuss recent literature regarding how impaired Ca^2+^ handling, dysfunctional Ca^2+^-dependent production of ROS/RNS, and structural/functional alterations in CRUs–mitochondria association could contribute to loss of muscle function, increased damage following muscle rupture, and reduced muscle mass and contractile function in inherited muscle diseases, sarcopenia, and disuse atrophy.

## 3. Altered Ca^2+^ Handling and Mitochondrial ROS Production in Inherited Forms of Skeletal Muscle Disease

### 3.1. Muscular Dystrophy

Muscular dystrophies (MDs) comprise a heterogeneous group of muscle diseases characterized by weakness, muscle wasting, and progressive muscle degeneration that can ultimately lead to an impairment of mobility and premature death. The most common MD is the Duchenne muscular dystrophy (DMD), a currently incurable inherited X-linked recessive muscle disorder that affects 1 in 3500 male births. DMD is caused by loss-of-function mutations in the gene encoding dystrophin, a 427 kDa structural protein located at the cytoplasmic face of the sarcolemma [94]. Dystrophin links actin filaments and microtubules of the cytoskeleton to the extracellular matrix through a group of proteins collectively known as the dystrophin-glycoprotein complex (DGC). Disruption of the DGC, due to the loss of both dystrophin and sarcoglycan proteins, results in a reduction in sarcolemma integrity/stability, which results in microtears in the sarcolemma during mechanical stress. Until they are repaired, microtears promote non-specific influx of Ca^2+^ (and other ions) across the membrane followed by pathological myoplasmic Ca^2+^ overload that triggers an array of intracellular mechanisms that lead to myofiber degeneration and death including: dysregulation of cytosolicCa^2+^ homeostasis, mitochondrial Ca^2+^ overload/damage, increased mitochondrial ROS production and oxidative stress, and activation of the Ca^2+^-dependent proteases [95,96] (Figure 1). While disruption of sarcolemmal integrity is a common feature among the different MDs, a comprehensive understanding of the mechanisms responsible for aberrant Ca^2+^ handling and increased oxidative stress that underlie the dystrophic phenotype are far from being fully understood. Currently, two main pathomechanisms are hypothesized to be responsible for abnormalities in myoplasmic Ca^2+^ levels in DMD: (1) excessive transmembrane Ca^2+^ influx and (2) enhanced SR Ca^2+^ leak through oxidized RyR1 Ca^2+^ release channels (Figure 1). Besides a non-selective Ca^2+^ influx through sarcolemmal microtears, a number of studies provide evidence for the involvement of more specific entry pathways through Ca^2+^-permeable channels in the plasma membrane. Early studies reported that a significant portion of the increased membrane permeability to external Ca^2+^ is mediated by stretch-activated Ca^2+^ channels [97]. Indeed, inhibition of these channels prevents the increased stretch-induced rise in intracellular Ca^2+^ observed in muscle fibers from *mdx* mice. Subsequent studies provided evidence for a modulatory role of enhanced SOCE in DMD. Prior to the identification of STIM1 and ORAI1 in SOCE, several studies reported that store-dependent Ca^2+^-permeable TRPC channel activity is upregulated in muscle fibers of *mdx* mice, and thus, contributes to increased levels of myoplasmic Ca^2+^ [98,99,100]. Subsequent studies found that increased STIM1/ORAI1-dependent SOCE also contributes to the enhanced Ca^2+^ influx observed in DMD (Figure 1), as both the expression and activity of STIM1/ORAI1-mediated SOCE are markedly enhanced in mouse models of MD [59,60] and the dystrophic phenotype of *mdx* is reduced by inhibiting Orai1-dependent SOCE [101].

In addition to sarcolemmal Ca^2+^ influx, increased SR Ca^2+^ leak through RyR1 channels represents an alternative proposed mechanism for altered Ca^2+^ handling in DMD. A series of studies from Marks and colleagues reported that loss of dystrophin (in *mdx* mice) or the DGC (in *Sgcb^-/-^* mice) is associated with hypernitrosylation of specific cysteine residues in RyR1, which lead to FKBP12 dissociation, destabilization of the RyR1 channel closed state, and increased RyR1-dependent SR Ca^2+^ leak [102,103]. The subsequent pathogenic rise in myoplasmic Ca^2+^ leads to mitochondrial Ca^2+^ overload and uncontrolled ROS/RNS production that further oxidizes/nitrosylates RyR1 in a destructive feed-forward cycle of Ca^2+^ leak and ROS/RNS production (Figure 1). The importance of increased oxidative stress as a key pathomechanism of muscle degeneration in DMD is supported by improvement of the dystrophic phenotype in *mdx* mice following treatment with N-actetylcysteine (NAC), a potent antioxidant [95]. Interestingly, an interplay between altered Ca^2+^ signaling and excessive mitochondrial ROS production was shown to be a key pathomechanism not only in MDs, but even in other muscle diseases such as malignant hyperthermia (MH) and central core disease (CCD) (see Section 3.2 for more details) (Figure 1). In fact, we previously reported that pre-treatment of mice with NAC for two months was able of reducing either anesthetic-triggered lethal hyperthermic episodes in mice lacking CASQ1 (CASQ1-null) [104] or mitochondrial damage and formation of *cores* in both heterozygous RYR1-Y522S [105] and CASQ1-null [106] mice.

A reduction in oxidative stress normalizes SR Ca^2+^ release by reducing the opening probability of destabilized RyR1 channels, thus interrupting the destructive feed-forward cycle of Ca^2+^ leak and ROS/RNS production and ameliorating the dystrophic phenotype. However, it remains unclear whether or not increased ROS/RNS levels modify proteins that mediate SOCE to augment Ca^2+^ entry and signaling. Likewise, it is unknown if inhibiting Ca^2+^ entry reduces oxidative stress. Future studies using mouse models to specifically inhibit SOCE in skeletal muscle will be needed to answer this important questionand, thus, validate SOCE as a potential therapeutic target to treat this incurable disease.

### 3.2. Malignant Hyperthermia and Central Core Disease

Aberrant Ca^2+^ handling and increased oxidative stress are pathomechanisms of the myopathic phenotype of two overlapping muscle disorders: MH susceptibility and CCD. MH is a potentially lethal inherited pharmacogenetic disorder characterized by a life-threatening hyperthermic reaction in susceptible individuals exposed to volatile/halogenated anesthetics (halothane, isofluorane, etc.) and/or succinylcholine, compounds commonly used during surgical procedures [107]. CCD, one of the most common inherited human congenital myopathies, is characterized by hypotonia, proximal muscle weakness, and delayed attainment of motor milestones [108,109]. An association between MH and CCD exists as muscle biopsies of some MH patients exhibit *cores* [110,111], amorphous central areas of muscle fibers lacking glycolytic/oxidative enzymes and mitochondria upon histological analysis [112]. In addition, CCD patients are at risk for hyperthermic episodes during exposure to MH-triggering agents (e.g., anesthetics) and muscle biopsies from some CCD patients exhibit increased susceptibility to contractures during in vitro caffeine-halothane contracture testing [113,114,115].

MH and CCD are inexorably linked to one another as most cases for both conditions are linked to mutations in the *RYR1* gene [116,117]. Association of gain-of-function mutations in the *RYR1* gene with MH (and some individuals with CCD) indicates that these disorders result, at least in part, from defective RyR1 function and Ca^2+^ regulation in skeletal muscle. MH-related mutations in RyR1 destabilize the SR Ca^2+^ release channel closed state, resulting in an increased susceptibility to opening in response to activators, SR Ca^2+^ leak, and mitochondrial Ca^2+^ uptake and subsequent ROS/RNS production [118]. In turn, RyR1 oxidative modifications (e.g., S-nitrosylation and S-gluthationylation) further destabilize the channel, and thus, further enhance RyR1 Ca^2+^ leak. This destructive feed-forward mechanisms of increased RyR1 Ca^2+^ leak and oxidative stress enhances RyR1 sensitivity to activation in MH that eventually leads to mitochondrial damage and the development of central *cores* in CCD [119] (Figure 1).

RYR1-Y522S knock-in mice [120] exhibit age-dependent development of central *cores* that occurs in conjunction with a mild myopathy, characterized by muscle weakness and mitochondrial damage, which together mirror key functional and structural abnormalities observed in human CCD patients [105,119,121]. MacLennan and colleagues proposed that the formation of *cores* in the center of the fiber, due to the Ca^2+^-dependent disruption of mitochondria, may represent a protective compensative response to chronic elevations in myoplasmic Ca^2+^ designed to protect the fiber from further Ca^2+^-induced damage [122]. However, it is worth to pointing out that in addition to *RYR1* gain-of-function mutations that enhance Ca^2+^ leak, some mutations in RyR1 linked to CCD reduce voltage-dependent SR Ca^2+^ release during EC coupling, a phenomenon referred to as “EC uncoupling” [123,124]. For instance, the I4897T CCD mutation in RyR1 (mouse RyR1 numbering) reduces the release of Ca^2+^ from the SR following skeletal muscle excitation (Figure 1). Individuals with this mutation exhibit muscle weakness with the presence of ultrastructural changes in muscle including disruption of the myofibrils, “Z-line streaming”, sarcomeric disorganization, predominance of fiber type I, and the lack of mitochondria in small areas of the fibers [125]. Interestingly, the reduction in SR Ca^2+^ release as the result of the I4897T mutation correlates with an increased mitochondrial damage-dependent oxidative stress, a feature that closely resembles that also observed in aged muscles (see Section 4 for more details). However, the structural abnormalities observed in I4897T mice are less severe than those observed in RYR1-Y522S mice, in line with excessive Ca^2+^ leak, larger degree of store depletion and higher oxidative stress displayed by the latter.

The critical pathogenic role of increased oxidative stress in the mitochondrial damage and formation of *cores* in CCD has been the subject of significant attention. Treatment of mice with an antioxidant (NAC) reduces mitochondrial damage and the development of *cores* in RYR1-Y522S mice, but not MHS [105,119]. Whether or not other mechanisms that control Ca^2+^ homeostasis in muscle are also involved in MH and CCD pathophysiology remains poorly understood. The reason might lie in the fact that for many years it was assumed that sustained elevations in myoplasmic Ca^2+^ are solely the consequence of increased RyR1-mediated SR Ca^2+^ leak/release. However, excessive RyR1 Ca^2+^ leak can lead to local SR depletion that activates SOCE to further enhance resting Ca^2+^ levels (see [126] for detailed discussion) (Figure 1). In support of this idea, muscle fibers/myotubes from RYR1-Y522S were shown to exhibit reduced SR Ca^2+^ content [118,127] and an increased rate of SOCE [128], suggesting increased STIM1 and ORAI1 expression/function. Consistent with this, increased SOCE activity was postulated to be an important mechanism for aberrant cytosolic Ca^2+^ dynamics in muscle biopsies of MH patients [61,62]. Nevertheless, further studies will be required to determine whether SOCE contributes to the pathogenesis of MH and CCD, and thus, if this pathway represents a potential therapeutic target.

## 4. Role of Altered Ca^2+^ Handling and Mitochondrial ROS Production in Loss of Muscle Mass and Reduced Contractility in Sarcopenia

Sarcopenia, age-related skeletal muscle decline, is a major national health problem. Sarcopenia is characterized by loss of muscle mass, lowered strength, increased susceptibility to fatigue, and reduced velocity of contraction [129,130]. The loss of muscle mass during aging is primarily due to reduced fiber number and size [131,132], degeneration of neuromuscular junctions as the result of stem cell depletion [133], and loss of motor units [134]. However, age-related muscle atrophy alone is not sufficient to account for the massive decline of muscle function and weakness observed during aging. Indeed, while muscle atrophy certainly contributes to muscle weakness in aging, the decline in muscle strength and increase in susceptibility to fatigue occur even before development of atrophy, consistent with a reduction in intrinsic muscle specific force production that is independent of muscle size or neuromuscular function. 

Dysregulation in Ca^2+^ handling and increased oxidative stress are two mechanisms proposed to contribute to age-dependent reduction in muscle specific force. In seminal studies conducted by Delbono and colleagues, age-dependent decline in intrinsic muscle force production was shown to involve a marked reduction in DHPR α1-subunit expression that results in a functional uncoupling of DHPR and RyR1 proteins in CRUs [135,136] (Figure 1). A reduction of both voltage sensor function and L-type Ca^2+^ current activity with aging was associated to an impaired voltage-dependent SR Ca^2+^ release and specific force production [137,138]. Boncompagni et al. (2006) proposed a slightly different explanation for the inefficient delivery of Ca^2+^ ions to the contractile elements in aged fibers [139]. This study found a progressive reduction in the number of CRUs in muscle biopsies from sedentary seniors (loss of about 40–50% of CRUs compared to muscles from young adults) as the cause of specific force loss in aging muscle. These findings were subsequently supported by studies in mice [93] and human muscle biopsies [140] that quantified mitochondrial association with CRUs: a significant reduction of both the total number/density of CRUs, mitochondria, and CRU–mitochondrial pairs in muscles from aged mice/humans. These structural alterations were accompanied by parallel reductions in SR Ca^2+^ release during EC coupling, impaired mitochondrial Ca^2+^ uptake, and increased levels of oxidative stress [93]. Similar results were observed in human biopsies where total mitochondrial number and CRU–mitochondrial pairs are higher in well-trained seniors who exercised regularly for the past 30 years compared to age-matched healthy sedentary seniors [140]. Interestingly, these age-dependent structural and functional alterations were almost prevented by regular endurance exercise when mice housed in cages with voluntary exercise wheels [141].Specifically, in this study, long-term exercise prevented and/or corrected age-dependent uncoupling of mitochondria from the EC coupling apparatus, thus preserving SR Ca^2+^ release during EC coupling, mitochondrial Ca^2+^ uptake, and reduced levels of oxidative stress. Thus, the correct positioning of mitochondria with respect to the SR is essential not only for correct Ca^2+^ handling, but also for proper maintenance of physiological levels of ROS/RNS. It is interesting to note that oxidative stress occurs both as a result of mitochondrial Ca^2+^ overload (as observed in DMD, MH, and CCD) (Figure 1) and when mitochondrial Ca^2+^ uptake is reduced, consistent with correct Ca^2+^ signaling being required for proper control of the redox state. Future studies will be required to elucidate the molecular mechanisms underlying the complex Ca^2+^-dependent regulation of mitochondrial ROS production.

A third mechanism proposed to contribute to an age-dependent reduction in intrinsic muscle specific force generation involves altered SR Ca^2+^ release function due to oxidative stress-dependent modification in RyR1, likely due to altered mitochondrial function. In line with this idea, Marks and colleagues reported an age-dependent increase in RyR1 oxidation/nitrosylation, FKBP12 dissociation, and SR Ca^2+^ leak [142]. As a result of this increased SR Ca^2+^ leak, peak electrically evoked Ca^2+^ release and muscle specific force production were reduced in muscle from aged mice [142]. 

Beside impaired EC coupling due to DHPR/RyR1 uncoupling, reduced CRUs and CRU–mitochondrial association, altered mitochondria structure/function, and increased nitrosylation-dependent RyR1 Ca^2+^ leak, a reduction in STIM1/ORAI1-mediated SOCE activity is also proposed tocontribute to age-dependent reduction in muscle specific force productionandincreasedsusceptibility to fatigue in aged muscle. In support of this idea, muscles from 2-year-old mice exhibit reduced SOCE and increased susceptibility to fatigue during high-frequency stimulation [143,144]. We recently reported that *extensor digitorum longus* muscles from 2-year-old mice exhibited an accelerated decline in force generation during high-frequency stimulation compared to that of muscles from 4-month-old mice [64]. Consistent with a reduced role for Ca^2+^ entry in aged muscle, removal of Ca^2+^ from the extracellular medium during repetitive high-frequency stimulation reduced contractility of muscles from young, but not aged, mice. These data are in line with findings of Thornton and colleagues who reported an inability of aged muscle to recover Ca^2+^ ions from the extracellular space via SOCE [63].

An age-dependent reduction in SOCE activity could lead to impaired SR Ca^2+^ refilling during prolonged muscle activity, and thus, lead to reduced Ca^2+^ availability within the SR to support force production during sustained activity. This idea is consistent with the fact that pharmacological and/or genetic inhibition of SOCE in skeletal muscle of young mice results in a reduction in force generation and increased susceptibility to fatigue [44,45,63,143]. However, the relative role of STIM1/ORAI1-mediated SOCE in age-related decline in muscle contractility remains controversial as other groups reported that: (1) the role of SOCE is marginal and/or absent in the maintenance of force generation during high-frequency repetitive stimulation [145] and (2) despite a significant reduction of STIM1 expression (~40%), SOCE activity is unaltered in muscle fibers from aged mice [146]. Clearly, additional studies are needed to fully elucidate the role of SOCE in muscle dysfunction during aging. 

## 5. Altered Mitochondrial Function and Ca^2+^ Homeostasis in Muscle Atrophy

Skeletal muscle atrophy reflects the loss of muscle mass as a result of muscle disuse (e.g., bed rest, limb immobilization or unloading, space flight, mechanical ventilation, or denervation) and certain pathological conditions (cancer, diabetes, sepsis) [147]. Muscle atrophy, commonly accompanied by a loss of muscle strength, results from a net effect of decreased protein synthesis and/or increased protein degradation, with proteolysis often being a dominant contributing factor [148]. Among the multitude of proteolytic systems identified, the calpain and caspase-3 systems are Ca^2+^-dependent signaling pathways involved in the dynamic process of protein degradation [149] (Figure 1). Specifically, Ca^2+^ alloserstically activates calpainand caspase-12 to activate caspase-3, which cleaves cytoskeletal proteins to disrupt myofilaments and facilitate protein degradation. In this regard, dysregulation in myoplasmic Ca^2+^ levels promotes atrophy in various muscle disuse animal models and pathological conditions [150,151]. For example, a ~20–30%increase in resting myoplasmic Ca^2+^ was observed in soleus muscle fibers following hindlimb unloading and reloading in rats [152]. In an in vitro model of cancer cachexia, exposure to proteolysis-inducing factor triggered a transient increase in myoplasmic Ca^2+^ level through activation of Ca^2+^ release via inositol 1,4,5 trisphosphate receptors, that was proposed to activate downstream caspase-mediated proteolytic pathways [153,154]. However, further studies are needed to establish a direct causative link between myoplasmicCa^2+^ activation of calpain-mediated downstream proteolytic pathways in muscle atrophy during cancer cachexia.

Beyond Ca^2+^ dysregulation, increased oxidative stress also promotes muscle atrophy, as administration of antioxidants can prevent or alleviate atrophic responses [155]. Of note, increased SR Ca^2+^ leak due to oxidative stress represents an important proposed mechanism for skeletal muscle atrophy. Specifically, Matecki et al. [156] reported that long-term mechanical ventilation results in S-nitrosylation and RyR1 Ser-2844 that leads to reduced Ca^2+^ spark activity during electrical stimulation of the diaphragm, which was mitigated by antioxidant treatment with Trolox to prevent RyR1 oxidation, SR Ca^2+^ leak and diaphragm weakness. Diaphragm dysfunction was also prevented by treatment with S107, a small molecule that stabilizes the association of FKBP12 with RyR1. Similarly, S107 administration attenuated RyR1 hyper-nitrosylation and skeletal muscle atrophy in chronically hypoxic animals due to exposure to high altitude [157]. Taken together, these studies suggest that increased oxidative stress, RyR1 post-translational modification and SR Ca^2+^ leak contribute to certain forms of muscle atrophy and weakness. 

In addition to oxidative stress-induced changes in RyR1 Ca^2+^ leak, oxidation of SERCA can also contribute to skeletal muscle atrophy and weakness in both humans and animals [158,159,160]. Qaisar et al. [159] reported that increased oxidative stress leads to reduced SERCA activity and mitochondrial dysfunction in SOD1-deficient mice. Consistent with this, CDN1163, an allosteric SERCA activator, restored SERCA activity, attenuated loss of muscle mass, and alleviated mitochondrial ROS production and oxidative damage [158]. Moreover, an increase of sarcolipin, a protein that regulates SERCA activity and muscle thermogenesis, coincided with reduced SERCA expressions and activity, as well as increased CASQ1 expression, lipid peroxidation, and mitochondrial ROS production in skeletal muscle of asthmatic patients [159]. Increased sarcolipin transcription and protein expression, along with increased phosphorylated Ca^2+^/calmodulin-dependent protein kinase II, were observed in atrophied muscles of mice following hindlimb immobilization [160]. Although measurements of myoplasmic, mitochondrial, and SR Ca^2+^ levels are needed, these studies suggest that increased oxidative stress signaling leading to alterations in Ca^2+^ homeostasis plays a significant role in certain forms of muscle atrophy and weakness.

Mitochondrial dysfunction is an additional key feature in the development and progression of skeletal muscle disuse atrophy (Figure 1). Mitochondrial alterations during disuse atrophy include reduced volume, disrupted morphology and dynamics (fusion and fission), and dysfunction including defective respiratory activity, reduced mitochondrial protein levels, and increased mitochondrial ROS production [161,162,163,164]. A critical role of increased oxidative stress due to mitochondrial dysfunction in muscle atrophy was demonstrated by amelioration of mitochondrial ROS production, muscle oxidative damage, and protease activation during disuse atrophy following treatment with the mitochondrial-targeted antioxidant peptide, SS-31 [165]. As a consequence of damage, mitochondria release apoptosis inducing factor (AIF) and cytochrome C into the cytosol, which activates caspase-3 to trigger myonuclear apoptosis and nuclear DNA fragmentation [166,167]. Indeed, denervation-induced muscle atrophy is attenuated in a double knockout model of Bax and Bak, which prevent the release of AIF and apoptosis. Together, these studies indicate that mitochondrial dysfunction leading to increased oxidative stress and activation of apoptotic pathways are important components of the signaling cascade that underlies disuse muscle atrophy.

A potential mechanism for mitochondrial dysfunction in muscle atrophy is that enhanced mitochondrial Ca^2+^ uptake could promote mitochondrial ROS generation that subsequently activates downstream muscle atrophy signaling pathways (Figure 1). In contrast to this idea, mitochondrial ROS production is increased in the absence of detectable changes in either myoplasmic Ca^2+^ concentration or mitochondrial Ca^2+^ uptake in *flexor digitorum brevis* fibers during denervation-induced muscle atrophy [168]. Electrical stimulation was able to promote mitochondrial Ca^2+^ uptake and reduce mitochondrial ROS production. Importantly, these effects were abolished by Ru360, an inhibitor of mitochondrial Ca^2+^ uptake, consistent with mitochondrial Ca^2+^ uptake being required to mitigate mitochondrial ROS production during denervation. As one possible mechanism, mitochondrial Ca^2+^ uptake could result in partial depolarization of the mitochondrial membrane potential, which would reduce mitochondrial ROS production by Complex I [169]. Consistent with these results, Mammucari et al. [170] reported that muscle atrophy following denervation was reduced by overexpression of MCU to enhance mitochondrial Ca^2+^ uptake. Additional work is needed to assess the relative impact of mitochondrial Ca^2+^ uptake and mitochondrial ROS production in other models of muscle atrophy (e.g., disuse atrophy).

## 6. Concluding Remarks and Future Directions

Over the past several years, substantial progress was made in elucidating exciting new insights into the role of impaired Ca^2+^ homeostasis and increased oxidative stress in the loss of muscle mass, increase in muscle damage, and reduction in muscle contractility that are characteristic of sarcopenia, disuse atrophy, and a wide range of muscle disorders including DMD, CCD, and heat/exercise-induced rhabdomyolysis. Nevertheless, precise molecular mechanisms are yet to be fully understood and a considerable number of unresolved issues and open questions remain to be addressed. 

This review provides a comprehensive overview of the literature supporting a role for dysfunctional Ca^2+^ handling and Ca^2+^-dependent mitochondrial ROS/RNS production in the decline of muscle function during aging, muscle atrophy, and the pathogenesis of several genetically inherited muscle disorders. These advances identify several signaling pathways and molecular mechanisms that represent potential new targets for the development of more effective therapies to treat a wide range of debilitating human myopathies.

## Abbreviations

AIF	apoptosis inducing factor
ATP	adenosine triphosphate
Ca^2+^	calcium
CCD	central core disease
CEU	Ca^2+^ entry unit
CRU	Ca^2+^ release unit
CASQ1	calsequestrin-1
DGC	dystrophin-glycoprotein complex
DMD	Duchenne muscular dystrophy
DHPR	dihydropyridine receptor
EC coupling	excitation-contraction coupling
ER	endoplasmic reticulum
MCU	mitochondrial Ca^2+^ uniporter
MD	musclular dystrophy
MH	malignant hyperthermia
RNS	reactive nitrogen species
ROS	reactive oxygen species
RyR1	ryanodine receptor type-1
SERCA	sarco/endoplasmic Ca^2+^ ATPase
SOCE	store-operated Ca^2+^ entry
SR	sarcoplasmic reticulum
STIM1	stromal-interacting molecule 1
TA	tubular aggregate
TAM	tubular aggregate myopathy
TCA	tricarboxylic acid
T-tubule	transverse tubule
